# Hydrogen peroxide in deep waters from the Mediterranean Sea, South Atlantic and South Pacific Oceans

**DOI:** 10.1038/srep43436

**Published:** 2017-03-07

**Authors:** Mark J. Hopwood, Insa Rapp, Christian Schlosser, Eric P. Achterberg

**Affiliations:** 1 Chemical Oceanography, GEOMAR Helmholtz Centre for Ocean Research Kiel, 24148 Kiel, Germany

## Abstract

Hydrogen peroxide (H_2_O_2_) is present ubiquitously in marine surface waters where it is a reactive intermediate in the cycling of many trace elements. Photochemical processes are considered the dominant natural H_2_O_2_ source, yet cannot explain nanomolar H_2_O_2_ concentrations below the photic zone. Here, we determined the concentration of H_2_O_2_ in full depth profiles across three ocean basins (Mediterranean Sea, South Atlantic and South Pacific Oceans). To determine the accuracy of H_2_O_2_ measurements in the deep ocean we also re-assessed the contribution of interfering species to ‘apparent H_2_O_2_’, as analysed by the luminol based chemiluminescence technique. Within the vicinity of coastal oxygen minimum zones, accurate measurement of H_2_O_2_ was not possible due to interference from Fe(II). Offshore, in deep (>1000 m) waters H_2_O_2_ concentrations ranged from 0.25 ± 0.27 nM (Mediterranean, Balearics-Algeria) to 2.9 ± 2.2 nM (Mediterranean, Corsica-France). Our results indicate that a dark, pelagic H_2_O_2_ production mechanism must occur throughout the deep ocean. A bacterial source of H_2_O_2_ is the most likely origin and we show that this source is likely sufficient to account for all of the observed H_2_O_2_ in the deep ocean.

H_2_O_2_ is found ubiquitously in natural surface waters^1^,^2^,^3^. Photochemistry^4^,^5^,^6^, biological activity^7^,^8^,^9^, and physical mixing^1^,^10^, are considered to be the dominant processes controlling H_2_O_2_ concentration in the surface ocean. H_2_O_2_ concentrations from 20–800 nM are reported in surface marine waters with a diurnal oscillation typically observed^11^,^12^,^13^. H_2_O_2_ is a reactive intermediate in the biogeochemical cycling of a range of elements in surface waters^14^,^15^. It also has direct effects on surface dwelling microorganisms as ambient H_2_O_2_ concentrations are sufficient to trigger oxidative stress in many marine phytoplankton species^16^,^17^.

Intriguingly, measurable concentrations of up to 6 nM H_2_O_2_ have been reported for the deep North Pacific^18^, far below the photic zone. The distribution of H_2_O_2_ throughout the deep ocean is however poorly constrained and thus the possible effects of H_2_O_2_ on biogeochemical cycles in these waters are presently difficult to assess. If H_2_O_2_ is found at low nM concentrations throughout the deep ocean this also raises questions about its origin. A photochemical source of H_2_O_2_ at these depths is not plausible so the foremost hypothesis is a dark biological source^19^,^20^ associated with bacterially mediated remineralisation of organic matter.

Here we report full depth profiles of H_2_O_2_ in three different ocean basins (Mediterranean Sea, South Atlantic and South Pacific Oceans) to determine whether measurable concentrations of H_2_O_2_ are found ubiquitously in the deep ocean. We also re-assess the robustness of the widely used flow-injection luminol based chemiluminescence method^12^,^21^,^22^ for the analysis of low (<10 nM) H_2_O_2_ concentrations in seawater.

## Results

### Verifying the accuracy of H_2_O_2_ measurements

As noted by Yuan and Shiller^18^, at low nM concentrations it is very difficult to establish conclusively that a measured, or ‘apparent’, concentration of H_2_O_2_ is real and not an artifact of sample handling or the result of compounds other than H_2_O_2_ causing a false positive signal. Potential systematic problems with light exposure, filtration and contamination from the atmosphere or de-ionized water residues exist. All of these potential H_2_O_2_ contamination sources were however eliminated. The use of opaque sample bottles and foil shields minimized background light. The ambient lighting used in our laboratory produced only a small increase in H_2_O_2_ concentrations even when seawater was deliberately exposed to light by removal of sample bottle lids and placement of samples directly below our 330 lumens light source (<0.6 nM min^−1^, which is also inclusive of any contamination arising from atmospheric H_2_O_2_). Filtration was not used for normal sampling purposes and all sampling apparatus was pre-conditioned with seawater to ensure no contamination from de-ionized water derived H_2_O_2_.

With respect to the possible detection of species other than H_2_O_2_, the H_2_O_2_ luminol based FIA method is expected to be robust under most circumstances^22^. Organic peroxidases typically produce a low luminol response relative to the equivalent molar concentration of H_2_O_2_ and are only present at <1 nM in surface seawater^23^. Yuan and Shiller^18^ for example found that the response of methyl hydroperoxide solution was 11% that of H_2_O_2_ (and this signal may have actually arisen from a H_2_O_2_ impurity). Some trace metal ions also cause a positive interference (Table 1)^22^. However, in oxygenated offshore seawaters the anticipated interferences should be minimal. Dissolved Fe (DFe) concentrations in the Atlantic for example rarely exceed 1 nM^24^,^25^ which is equivalent to an apparent H_2_O_2_ signal of <0.5 nM. The response of Fe(II) is however stronger than that of Fe(III) (Table 1). At low O_2_ concentrations the oxidation rate of Fe(II) to Fe(III) is reduced such that high (nM) concentrations of Fe(II) can be found in the water column^26^. For example, in the Southern Ocean Sarthou *et al*.^27^ measured Fe(II) half-lives with respect to oxidation of 3–11 min in surface seawater (at pH 8.0–8.1 and O_2_ 234–353 μM), whereas Scholz *et al*.^28^ determined that the apparent Fe(II) half-life in bottom water of the Peruvian oxygen minimum zone (OMZ) was 16–18 h (at pH 7.6, O_2_ <0.5 μM). Outside OMZs the half-life of Fe(II) in seawater is thus expected to be sufficiently short that the delay between sample collection and analysis (typically 10 min − 1 h) should be sufficient for Fe(II) to decay to undetectable (low pM) concentrations^29^. Furthermore, as an apparent H_2_O_2_ signal arising from mainly non-H_2_O_2_ components is deducted from our measured H_2_O_2_ concentrations (see below), this should include any residual concentration of Fe(II). However, accounting for Fe(II) remains problematic in OMZs where nM Fe(II) concentrations are present during analysis.

**Figure 1:**
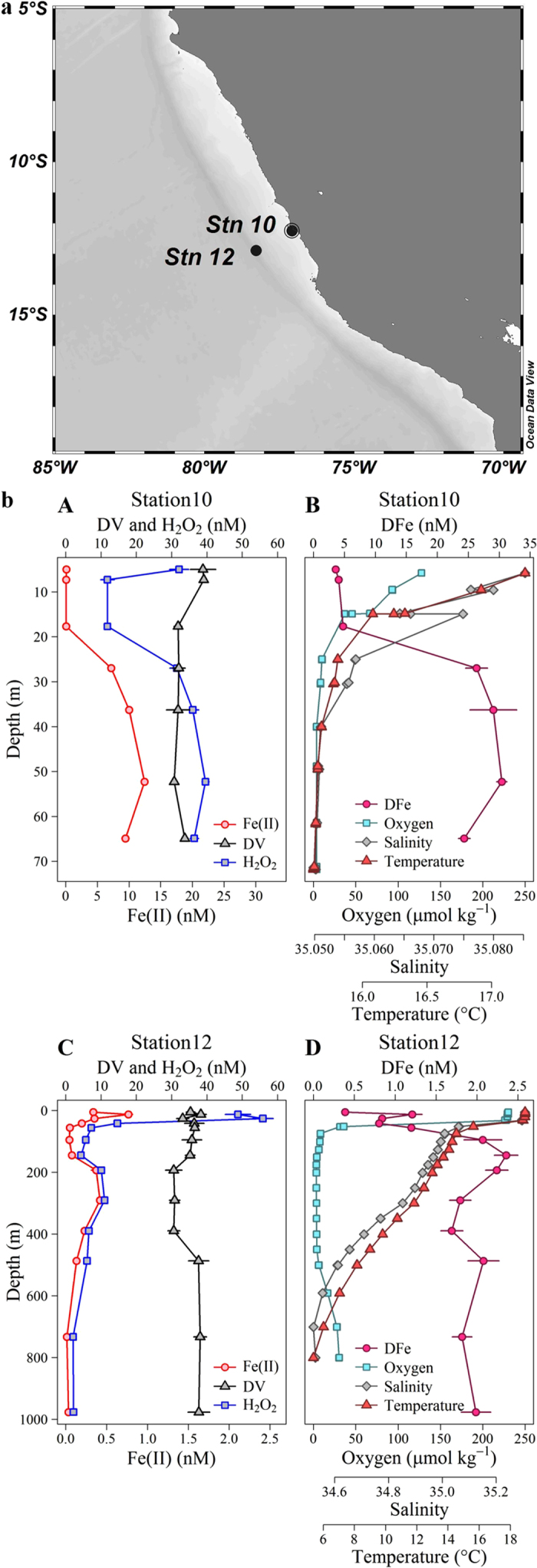
(**a**) Location of a shallow (Station 10) and offshore (Station 12) station within the Peruvian oxygen minimum zone (OMZ), plotted in Ocean Data View^54^. (**b)** H_2_O_2_ concentrations (after deduction of an aged seawater blank) and concentrations of potentially interfering trace metals at two stations in the OMZ. A Station 10 H_2_O_2_, dissolved (<0.2 μm) V (DV), Fe(II). B Station 10 Salinity, temperature, dissolved Fe (DFe), dissolved O_2_. C Station 12 H_2_O_2_, DV, Fe(II). D Station 12 Salinity, temperature, DFe, dissolved O_2_. (**b**) made in R version 3.2.3 (2015), GWDG Göttingen, Germany, http://ftp5.gwdg.de/pub/misc/cran/.

It is also particularly difficult to assess the effect of dissolved V (DV) on apparent H_2_O_2_ concentrations as less data are presently available on the distribution of reduced V species in the marine environment. Vanadium exhibits a relatively uniform concentration profile in the ocean with typical dissolved concentrations of 30–40 nM and a dominant oxidation state of V(V)^30^,^31^. Yet under reducing conditions a fraction of DV may exist as V(IV). Nanomolar concentrations of V(IV) have indeed been observed in sub-oxic and coastal waters^32^. As is the case for Fe(II) and Fe(III), any apparent H_2_O_2_ signal arising from V(IV) in oxic seawater (as per Table 1) should be accounted for by the deduction of an aged seawater blank from all measurements. But, as is the case for Fe(II), V(IV) interference may still be problematic in OMZs due to potentially enhanced concentrations which are subject to change as the sample is handled and analyzed. We present two depth profiles of DV in the S Pacific (Fig. 1) in order to assess the potential for this interference to occur within the vicinity of a coastal OMZ.

Trace metal samples were collected and analysed for Fe(II), DFe and DV at two stations (Stations 10 and 12, Fig. 1(a)) in the Peruvian OMZ. Oxygen depletion to <2 μmol kg^−1^ was evident in the water column at both stations (Fig. 1(b)). Consistent with results reported elsewhere along the Peruvian coastline^26^,^33^,^34^ low O_2_ waters below the surface mixed layer were enriched in DFe with the majority of DFe present as Fe(II). Up to 30 nM DFe was present at the shallow station (Station 10) and up to 2.4 nM at the offshore station (Station 12). Fe(II) concentrations were up to 12 nM and 0.77 nM at the shallow and offshore stations, respectively (Fig. 1(b)). The apparent H_2_O_2_ signal at these stations closely followed Fe(II) concentrations at all depths below 20 m, consistent with the interference demonstrated in aged seawater (Table 1). At both stations DV profiles exhibited a decline at intermediate depths associated with low O_2_ concentrations (Fig. 1(b)). The solubility of V(V) in seawater exceeds that of V(IV), thus a decline in DV concentration is expected under reducing conditions when V(IV) is formed^35^. Only a relatively small dissolved V(IV) concentration, ~1 nM, would be required to produce a nM apparent H_2_O_2_ signal (Table 1). Whilst we cannot determine the concentration of dissolved V(IV) present, it remains possible that this ion is a problematic interference with the luminol method for determination of H_2_O_2_ in coastal OMZs. It is not possible to correct for this interference as the concentration of reduced species (both Fe(II) and V(IV)) cannot be quantified with sufficient accuracy at the exact time H_2_O_2_ concentrations are measured. An aged (1–2 weeks old) seawater blank accounts for the presence of stable interfering species, but not unstable interfering species such as Fe(II) and V(IV)- the concentration of which must be known at the time H_2_O_2_ is measured if a correction is to be made. This difficulty arises, not only because of logistical constraints in coordinating the simultaneous measurement of multiple redox sensitive variables, but also because the measurement of Fe(II) via luminol itself is subject to a V(IV) interference^36^ and the chemiluminescence response of luminol to some ions, including Fe(II), is non-linear^37^. As an additional precaution we have therefore excluded all stations from our H_2_O_2_ dataset where Fe(II) exceeded 0.3 nM at multiple depths (Fe(II) was measured at all stations in the S Atlantic and S Pacific with a detection limit of ~0.2 nM, data not shown).

**Figure 2:**
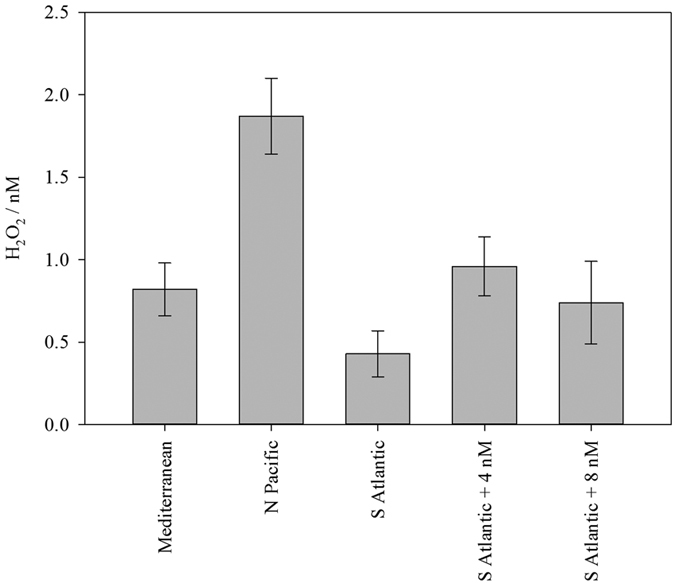
Apparent H_2_O_2_ measured after catalase treatment of different aged seawater samples . Mean (n = 4, ± standard deviation) H_2_O_2_ determined by flow injection analysis with no deduction made to account for positive interference from species other than H_2_O_2_. South Atlantic seawater was spiked (+4 and 8 nM H_2_O_2_) and re-analyzed to verify the effectiveness of catalase addition.

Some insight into what an apparent H_2_O_2_ signal is can be gained from use of the enzyme catalase, which removes H_2_O_2_ from solution. After surface seawater was aged in the dark at 6 °C for >2 weeks a variable apparent H_2_O_2_ signal of >1 nM could still be observed in all surface seawater tested. An apparent H_2_O_2_ signal of 0.4–1.9 nM was still measured after treatment with catalase at 25 °C for 1 h (Fig. 2). It is plausible that a residual, measurable concentration of H_2_O_2_ remained in seawater after catalase addition. Yet, if this residual H_2_O_2_ concentration depended primarily upon enzyme efficiency, we would expect to have observed the same residual H_2_O_2_ concentration in every catalase treated seawater sample. The residual signal however varied between water samples (Fig. 2). This indicated that the residual signal arose mainly from components other than H_2_O_2_.

As a further test to determine if the apparent H_2_O_2_ signal could actually have arisen from H_2_O_2_, we used the oxidation rate of Fe(II) in de-oxygenated seawater. Rate constants are available for the oxidation of Fe(II) via H_2_O_2_ in seawater (Equation (1)) at any environmentally relevant pH, temperature and salinity^38^,^39^. Thus, by comparing the oxidation rate of Fe(II) spiked into catalase treated and non-catalase treated aged seawater, the concentration of H_2_O_2_ present in aged seawater was estimated. The apparent H_2_O_2_ signal determined for aged S Atlantic seawater ranged from 0.43–0.96 nM after the addition of catalase (Fig. 2). When a 5 nM Fe(II) spike was added to this seawater, after de-oxygenating and treatment with catalase, the Fe(II) signal was stable for in excess of 1 h. Whereas, without catalase a 1.8 nM decrease was observed (in 1 h). Using the rate constant log k 4.17 M^−1^ s^−1^ (determined at 10 °C and pH 8.17^39^ matching our experimental conditions of 10.4 °C and pH 8.2) and assuming a pseudo-first order reaction (with constant [H_2_O_2_]) according to Equation (1), we determined the theoretical decline in Fe(II) concentration.


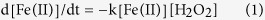


Assuming the decrease in Fe(II) concentration in non-catalase treated aged seawater could be attributed exclusively to oxidation by H_2_O_2_, the H_2_O_2_ concentration in aged S Atlantic seawater prior to catalase treatment was 7 nM. No change was observed in Fe(II) concentration after catalase treatment. This suggested that catalase effectively lowered the initial 7 nM H_2_O_2_ to no more than a few hundred pM. If H_2_O_2_ were actually present in solution at the apparent concentrations measured (0.4–1.9 nM, Fig. 2), 0.4 nM of H_2_O_2_ should have resulted in a decline of 0.1 nM Fe(II) over 1 h, and 1.0 nM of residual H_2_O_2_ should have resulted in a decline of 0.25 nM Fe(II). The absence of Fe(II) decay was consistent with our interpretation of the residual post-catalase apparent H_2_O_2_ signal (Fig. 2) as being due to components other than H_2_O_2_. For the purposes of calculating H_2_O_2_ concentration in seawater, the residual signal after aging and catalase treatment should therefore be treated as a blank. The detection limit of the method was therefore (3 standard deviations of the blank) 0.4–0.8 nM, similar to the typical blank of 0.6 nM reported elsewhere using similar apparatus^5^,^40^. This was higher than the 0.1–0.3 nM detection limit if the signal arising only from the reagent and background light/electrical noise was considered.

### H_2_O_2_ concentrations in the Mediterranean Sea and South Atlantic

Depth profiles of H_2_O_2_ were collected in the Mediterranean Sea (69 stations), S Atlantic (14 stations) and S Pacific Oceans. As outlined above, H_2_O_2_ measured at all 13 S Pacific stations and 30 S Atlantic stations was unreliable because of high (>0.3 nM) Fe(II) concentrations at multiple depths associated with coastal OMZs (Fig. 1). These stations are excluded and not discussed further.

**Figure 3:**
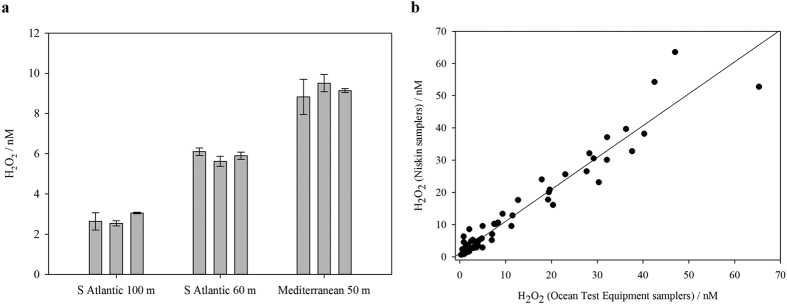
(**a**) Replicate apparent H_2_O_2_ measurements using 3 separate samplers deployed at the same depth. Mean (n = 4 injections) and standard deviation of apparent H_2_O_2_ shown. Apparent H_2_O_2_ included a signal arising from components other than H_2_O_2_ in solution. (**b**) Comparison between H_2_O_2_ samples from two sub-surface sampling methods. Ocean Test Equipment samplers mounted on a powder coated sampling rosette with a Kevlar cable, and Niskin samplers with internal metal components on a standard stainless steel rosette (n = 67 pairs) deployed at the same depths along the South Atlantic transect (December 2015, line of best fit plotted R^2^ = 0.94).

The reproducibility of the apparent H_2_O_2_ signal when sampling triplicate samplers from the same depth (Fig. 3(a)) verified that the signal was not subject to random contamination. A comparison of H_2_O_2_ measurements using equipment that was, or was not, trace metal clean (OTE and Niskin samplers with internal stainless steel components), indicated a small (mean 1.2 nM based on linear intercept, R^2^ = 0.94) overestimation of H_2_O_2_ concentration (Fig. 3(b)) when water was collected from Niskin samplers with metal components. The difference (n = 67 pairs) was statistically significant (P < 0.001, Wilcoxon Signed Rank Test – a paired test used here because it does not require a normally distributed population) and remained statistically significant both when the 3 highest concentrations were excluded (n = 64 pairs, P < 0.001) and when only concentrations <5.0 nM were compared (n = 40 pairs, P < 0.001).

**Figure 4:**
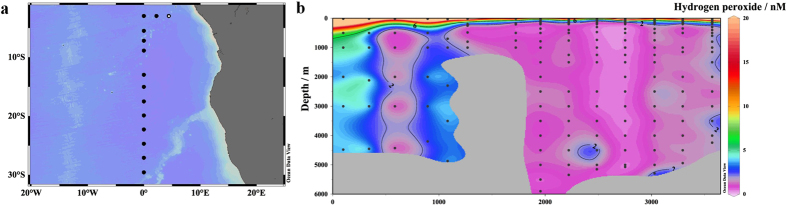
(**a)** GEOTRACES GA08 transect proceeding northeast-south in the South Atlantic, December 2015 (**b)** H_2_O_2_ concentrations in the South Atlantic plotted as distance along section shown (km) from northeast (left) to south (right). The first station (left) is marked on (**a**). Plotted in Ocean Data View^54^.

**Figure 5:**
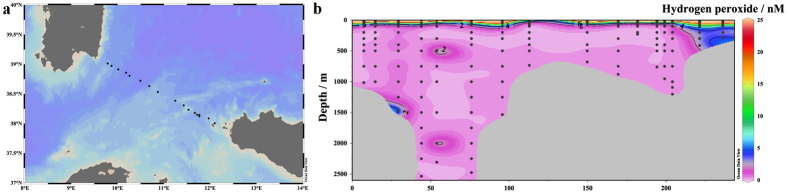
(**a**) Ocean Certain western Mediterranean transect between Sardinia and Sicily, August 2015 (**b**) H_2_O_2_ concentrations along the transect plotted as distance along section shown (km) from northwest (left) to southeast (right). Plotted in Ocean Data View^54^.

Proceeding from north-east to south-west along the S Atlantic transect, H_2_O_2_ was elevated at four of the first five stations throughout the water column, separated by a station with low H_2_O_2_ concentrations (Fig. 4) which were more typical of stations observed further south. These elevated concentrations may be associated with the OMZ, but this is difficult to confirm due to high (>0.3 nM) Fe(II) concentrations within the OMZ along the shelf (at excluded stations, not shown) that interfered with the method for H_2_O_2_ analysis (Table 1). In the Mediterranean (Fig. 5) the vertical profile of H_2_O_2_ was very similar to that observed at the southern-most stations in the S Atlantic. High (>4 nM) H_2_O_2_ was normally confined to approximately the uppermost 100 m. A notable exception was however found in shallower waters (<500 m) for example off the coast of west Sicily (Fig. 5) where the gradient in H_2_O_2_ concentration declined more slowly with depth and elevated (at least 2–5 nM) H_2_O_2_ was found throughout the water column.

**Figure 6:**
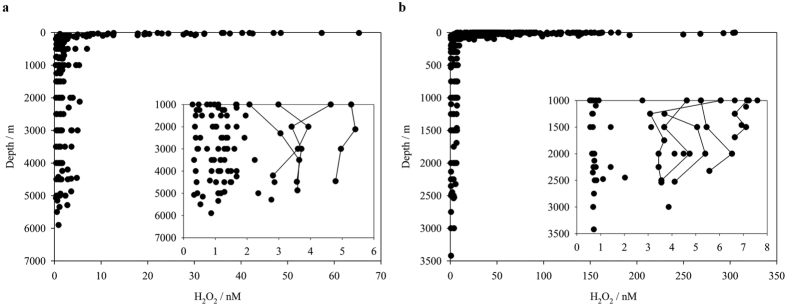
(**a**) H_2_O_2_ across all South Atlantic stations sampled. Only samples collected using trace metal clean Ocean Test Equipment samplers (181 data points, excluding 99 below detection limit) are shown (same data as Fig. 4b). Inset H_2_O_2_ at, or below, 1000 m (83 datapoints, excluding 80 below detection limit). For stations with multiple samples beneath 1000 m, profiles are connected if H_2_O_2_ exceeded 2.9 nM at any depth. (**b**) H_2_O_2_ across all Mediterranean stations. All sub-surface samples were collected from Niskin samplers free from internal metal components on a stainless steel cable (total 482 data points-including those shown in Fig. 5b, excluding 199 below detection limit). Inset H_2_O_2_ at, or below, 1000 m only (60 datapoints, excluding 64 below detection limit). For stations with multiple samples beneath 1000 m, profiles are connected if H_2_O_2_ exceeded 2.9 nM at any depth.

H_2_O_2_ concentrations in the surface ocean correlate strongly with irradiance^5^ and thus vary both diurnally and seasonally. Modest variation in temporally different reported concentrations in the same region is therefore expected. Our two most extensive datasets, for the S Atlantic (Fig. 6(a)) and the western Mediterranean (Fig. 6(b)), exhibited surface water concentrations of H_2_O_2_ with similar ranges to those reported by others^5^,^12^. Price *et al*.^12^ for example measured H_2_O_2_ concentrations ranging from 34–143 nM (mean 91 ± 29 nM) in surface waters from the Straits of Sicily, whereas here we report concentrations with a range 24–81 nM (mean 43 ± 22 nM).

## Discussion

A sharp decline in H_2_O_2_ concentration with depth was illustrated within the datasets as a whole (Fig. 6) consistent with the confinement of the dominant H_2_O_2_ source, direct photochemistry, to the surface ocean and H_2_O_2_ concentrations thus dropping rapidly below the thermocline^1^. Our depth profiles of H_2_O_2_ suggested that the source of H_2_O_2_ in the deep ocean is pelagic with no obvious benthic sources evident at depth in the S Atlantic or Mediterranean (Figs 4(b) and 5(b)). A H_2_O_2_ source may be associated with the OMZ in the Angolan Basin (Fig. 4(b)), but this is difficult to confirm given our exclusion of shelf stations from the S Atlantic dataset and the uncertainty associated with false positive interferences from trace metals within the OMZ (Fig. 1).

The H_2_O_2_ concentrations observed in deep (>1000 m) waters at some stations in both the S Atlantic and Mediterranean were within the range 1–6 nM previously reported in the N Pacific^18^. However, the mean concentrations measured here are lower with the majority of measurements at >1000 m depth in both the Mediterranean and S Atlantic below detection (<0.4−0.8 nM). There is thereby moderate uncertainty associated with determining the mean deep (>1000 m) H_2_O_2_ concentration as this depends on how the datapoints below detection are treated. Using the most conservative approach, where we consider that datapoints below detection may take any value between 0 and the detection limit, the mean H_2_O_2_ concentration for the four transects where sufficient data points are available (Table 2) ranges from 0.25 ± 0.27 to 2.9 ± 2.2 nM. The mean H_2_O_2_ concentration >1000 m globally is therefore likely within the range 0.25–2.9 nM.

Unfortunately the rate of H_2_O_2_ decay has not been determined at *in situ* temperature and pressure for non-surface marine waters. Yet, when Yuan and Shiller^18^ incubated deep seawater (from 1500 and 5000 m in the N Pacific) at 21 °C (at which temperature most H_2_O_2_ decay experiments are reported) the observed decay constant, which is assumed to be first order, was 0.003 h^−1^ corresponding to a half-life of 230 h. This was slower than the decay measured in N Pacific surface waters where rate constants ranged from 0.004–0.02 h^−1^, corresponding to half-lives of 33–170 h^18^,^41^,^42^. As H_2_O_2_ decay is considered to be primarily microbially mediated^43^ decay constants in deep seawater would be expected to scale approximately with microbial activity^18^. Anticipated decay rates in the deep ocean would thereby be at least two orders of magnitude smaller than those in surface waters^18^,^44^,^45^. The H_2_O_2_ decay rate constant measured at 1500 and 5000 m in the N Pacific is thereby quite high (14–72% of that measured in the same region for surface waters), which may imply that an inorganic mechanism also contributes to the observed decay rate^18^. There are several uncertainties here as it is unknown to what extent the mechanism for H_2_O_2_ decay in the deep ocean remains primarily enzymatic, as it is in surface waters, and it is also unclear whether a rate constant determined at 1 atm pressure and 21 °C can be extrapolated to *in situ* conditions at >1000 m depth.

None the less, if this rate constant is applicable to the deep ocean *in situ* and, as we suggest here, H_2_O_2_ is found throughout the deep ocean at a mean concentration of 0.25–2.9 nM, H_2_O_2_ must be produced at a rate of 0.8–9 pM h^−1^ to balance the measured decay. Reported rates of dark H_2_O_2_ production vary in the surface ocean, but two studies in different environments observed very similar ranges: peak surface dark H_2_O_2_ production rates for unfiltered coastal and Sargasso seawater are reported as 0.8–2.4 nM h^−1^ 46 and 1–3 nM h^−1^ 19, respectively. The supposed dark production rate of H_2_O_2_ in the deep ocean would thereby have to be 0.03–1.1% of that found in surface waters. Measurements of leucine incorporation suggest that bacterial activity at 1000 m depth is approximately 0.8% of that at the surface^47^. So, whilst not conclusive evidence, this calculation does at least suggest that a dark production mechanism, similar to that observed in surface waters^19^,^46^, could feasibly account for all of the H_2_O_2_ present in the deep ocean.

## Conclusions

Several trace metal species cause positive interferences with the widely used luminol-chemiluminescence method for H_2_O_2_ analysis in seawater. The majority of a residual 0.43–1.9 nM apparent H_2_O_2_ signal found in all aged, catalase treated seawater sampled cannot be attributed to H_2_O_2_ and should therefore be treated as a blank, which raises the detection limit of the method to 0.4–0.8 nM. Within the vicinity of OMZs, the concentration of Fe(II), and possibly also V(IV), is sufficiently high to dominate the apparent H_2_O_2_ signal in seawater below the thermocline. These interferences from transient species cannot presently be removed with great certainty.

After accounting for the apparent H_2_O_2_ signal arising from interfering ions, H_2_O_2_ appears to be present at very low nM concentrations in deep (below 1000 m) seawaters with the mean concentration varying across the different transects sampled: 2.9 ± 2.2 nM (Mediterranean, Corsica-France), 0.25 ± 0.27 nM (Mediterranean, Balearics-Algeria), 0.55 ± 1.3 nM (Mediterranean, Sardinia-Sicily) and 0.90 ± 1.2 nM (S Atlantic, Angola Basin). The source of this H_2_O_2_ remains uncertain, yet the observed distribution is consistent with a pelagic source. Estimates of deep ocean H_2_O_2_ decay rates and bacterial activity suggest that dark, bacterial production of H_2_O_2_ is sufficient to explain all H_2_O_2_ present at these depths.

## Methods

### Cruise work

Cruise work was conducted onboard RV Minerva Uno (Ocean Certain, August 2015, western Mediterranean Sea), FS Sonne (SFB 754, October 2015, South Pacific Ocean) and FS Meteor (GEOTRACES, November-December 2015, South Atlantic Ocean). All sample and reagent bottles for H_2_O_2_ analyses (opaque high density polyethylene (HDPE), Nalgene) were pre-cleaned by soaking sequentially in Mucasol detergent (Sigma-Aldrich) for 1 day and 1 M HCl for 1 week with 3 de-ionized water (18.2 MΩ cm^−1^, Milli-Q, Millipore) rinses after each stage. Bottles were then dried in a laminar flow hood and stored in re-sealable plastic bags until required. Seawater for the analysis of H_2_O_2_ was collected using various methods to assess sampling artifacts. Seawater was either collected: from surface waters using a plastic bucket on a nylon line twice rinsed with surface seawater before sample collection, from a towfish which continuously pumped surface seawater through plastic tubing into a clean laboratory on deck, from trace metal clean Ocean Test Equipment (OTE) samplers mounted on a powder coated sampling rosette with a Kevlar conducting cable complying with GEOTRACES specifications for trace metals, or from Niskin samplers (with, and without, internal metal components) mounted on a standard stainless steel sampling rosette with a stainless steel conducting cable. In all cases the HDPE sample bottles were rinsed 3 times with seawater, filled gently to overflowing, and sealed with no headspace. Samples were not filtered. For OTE and Niskin samplers, seawater was collected immediately after dissolved gas collection. Seawater was collected straight from the spigot of the samplers to minimize light exposure. All samples were analyzed within 2 h of sampling from the OTE or Niskin samplers. For surface samples, the time delay between sample collection and analysis for H_2_O_2_ was approximately 10 min.

### Chemical analysis

H_2_O_2_ concentrations were determined via flow injection analysis (FIA) using the Co(II) catalyzed oxidation of luminol, which is the most commonly used analytical method in seawater^12^,^21^,^22^. A FIA system was assembled using two 10-port valves (Valco, Vici), a photonmultiplier tube (PMT, H9319-11, Hamamatsu), a glass flow cell with a mirrored base (Waterville Analytical Products) and a peristaltic pump (MiniPuls 3, Gilson). The flow cell, PMT and valve were arranged as per Yuan and Shiller^22^, but with two alternating reagent loops such that one loop was loading and one loop unloading, with the same flow rates (2.5 mL min^−1^ sample and 1.4 mL min^−1^ reagent) and target sample loop internal volume (60 μL) used^22^. It was verified that this configuration produced close to optimal chemiluminescence peak height responses by testing the effect of small changes to flow rate and tubing lengths (reagent loop and, separately, the length of tubing between the valve and detector) on the peak height produced for a 50 nM standard addition to aged seawater. H_2_O_2_ concentrations were derived from mean peak height and are reported as the mean (±standard deviation) of 4 injections. The peak height response was always linear in the range 0–100 nM (R^2^ typically > 0.99 and always > 0.98). The PMT was secured inside an electrical box to minimize background light, and all reagent and sample tubing was opaque (black PTFE, Global FIA, 0.8 mm internal diameter) except peristaltic pump tubing (PVC, Gradko).

The H_2_O_2_ reagent was prepared by adding the following reagents to aged de-ionized water (stored at room temperature for >24 h in the dark) and making up to a total volume of 2 L: 0.54 g luminol (98%, ROTH), 44 g potassium carbonate (reagent grade, ROTH), 7.8 mL Co(II) solution (1000 ppm standard in 2% by volume HNO_3_, ROTH) and approximately 15 mL HCl (Trace Metal grade, Fisher Scientific) to adjust the pH of solution to 10.2^22^. Once mixed, H_2_O_2_ reagent was stored at approximately 6 °C for in excess of 24 h (to maximize the luminol response^22^) and then allowed to warm to room temperature for 6–12 h before use. Reagent remaining unused after 3 days was discarded. Between stations/experiments the FIA apparatus was rinsed with 0.1 M HCl followed by de-ionized water. To ensure the complete removal of acid and de-ionized water, an aged seawater blank was always run prior to re-commencing analysis. Calibrations were undertaken between stations, and with new reagent batches, using at least 6 standard additions of H_2_O_2_ (TraceSelect, Fluka) to aged (unfiltered) seawater obtained from >1000 m depth (stored at room temperature in opaque HDPE bottles). H_2_O_2_ was sequentially diluted to create stock solutions of 100 mM and 100 μM using de-ionized water. The use of narrow-necked, opaque HDPE sample bottles, aluminium foil shields over the apparatus and reduced lighting in the laboratory minimized the potential for undesired photochemistry during sample analysis.

### Experiments to verify the accuracy of H_2_O_2_ analysis

Mediterranean, Atlantic and Pacific seawaters were stored in the dark at 6 °C for >2 weeks for assessment of the baseline signal from a mixture of luminol reagent and seawater in the absence of high H_2_O_2_ concentrations. This aged seawater was then spiked with a freshly prepared solution of bovine catalase (Sigma) to a final enzyme concentration of 1–3 units mL^−1^, and allowed to stand with gentle mixing at 25 °C (to optimize enzyme performance) for 1 h. H_2_O_2_ spikes of 4 and 8 nM were also added to Atlantic seawater and treated identically to verify the effectiveness of the enzyme addition. The oxidation rate of a 5 nM Fe(II) spike in low O_2_ seawater was used to estimate the concentration of residual H_2_O_2_ in aged, catalase treated seawater. Fe(II) was spiked into aged seawater with low O_2_ in the presence and absence of catalase under a N_2_ atmosphere. After seawater was aged in the dark at 6 °C for 3 days, N_2_ was bubbled through seawater for >2 h. An Fe(II) spike was then added and the concentration of Fe(II) measured continuously for a period of >1 h. Fe(II) concentration was measured via FIA using luminol^48^,^49^,^50^ with a dual Fe(II)/H_2_O_2_ system^51^ assembled using the same components as the H_2_O_2_ system (see above). FIA was conducted in a laminar flow hood using the same procedures for handling reagent and seawater solutions as per the H_2_O_2_ analysis. Fe(II) reagent solution was made with 0.26 g luminol and 1.06 g potassium carbonate in 10 mL de-ionized water and stored overnight at 6 °C to ensure complete dissolution. This was then added to a 2 L solution of de-ionized water containing 80 mL ammonium hydroxide (Trace Metal grade, Fisher Scientific), to which approximately 22 mL HCl (Trace Metal grade, Fisher Scientific) was added to adjust the final pH to 10.1. The mixed reagent was allowed to stand for >24 h prior to use to maximize the luminol response^37^.

The interference with the H_2_O_2_ luminol chemiluminescence method from Fe(II), Fe(III) and V(IV) was investigated by spiking solutions of these ions into S Atlantic seawater that had been aged in the dark. Fe(II) additions were made into both low (aged, no H_2_O_2_ spike) and high (aged, spiked to 42 nM) H_2_O_2_ Atlantic seawater as the luminol response to some interferences may be non-linear^36^,^37^. Fe(II), V(IV) and Fe(III) stock solutions were made from ammonium Fe(II) hexahydrate (99%, Sigma Aldrich), vanadyl sulfate hydrate (99.99%, Sigma Aldrich) and Fe(III) chloride hexahydrate (>99.99%, Sigma Aldrich), respectively. Primary stocks were acidified using 1 mL HCl (Trace Metal grade, Fisher Scientific) per L solution and used on the day of preparation to minimize trace metal oxidation state changes.

To assess the potential for *in-situ* metal concentrations to interfere with H_2_O_2_ measurement in samples from OMZs, samples for trace metal analysis were collected from OTE samplers on a Kevlar wire adhering to GEOTRACES standards for trace metal sample collection (FS Sonne, October 2015, South Pacific). Sub-sampling was conducted under N_2_ gas (purity > 99.999%) in a metal free clean container where the air was filtered. Samples for Fe(II) analysis were collected first and analysed immediately (analysis complete within 22 min of sub-sampling OTE samplers) without filtration as above. Dissolved seawater samples for trace metals (filtered with 0.2 μm, Acropack 500) were collected in 125 mL low density polyethylene (Nalgene) bottles (pre-cleaned according to GEOTRACES protocols: 1 day in Mucasol detergent, 1 week in 1.2 M HCl, 1 week in 1.2 M HNO_3_ with 3 de-ionized water rinses after each stage) and stored double sealed in plastic bags. Samples were acidified in a laminar flow hood within 1 day of collection (140 μL Optima grade HCl, Fisher Scientific) and then stored acidified for >6 months. Samples were buffered to pH 6.4 using a 1.5 M ammonium acetate buffer before loading onto a WAKO resin^52^ for off-line pre-concentration (SeaFAST, Elemental Scientific Inc.). The buffer was prepared using ammonium hydroxide solution (Optima grade, Fisher Scientific) and acetic acid (Optima grade, Fisher Scientific) in de-ionized water adjusted to pH 8.5. Elution was performed in 1 M distilled HNO_3_ (distilled using a sub-boiling PFA distillation system (DST-1000, Savillex) from SPA grade HNO_3_ (Romil)). Pre-concentrated samples were analysed by high resolution inductively coupled plasma-mass spectrometry (HR-ICP-MS, ELEMENT XR, ThermoFisherScientific) using isotope dilution for Fe and standard addition for V^53^. Measurement of SAFe GEOTRACES reference water S produced concentrations of V 33.6 ± 1.1 nM (no consensus value available) and Fe 0.091 ± 0.004 nM (consensus value: 0.093 ± 0.008 nM).

## Additional Information

**How to cite this article:** Hopwood, M. J. *et al*. Hydrogen peroxide in deep waters from the Mediterranean Sea, South Atlantic and South Pacific Oceans. *Sci. Rep.* **7**, 43436; doi: 10.1038/srep43436 (2017).

**Publisher's note:** Springer Nature remains neutral with regard to jurisdictional claims in published maps and institutional affiliations.

## Figures and Tables

**Table 1:** Apparent H_2_O_2_ signal produced in aged South Atlantic seawater when spiked with a selection of trace metal species.

Metal ion	Spike added	Measured H_2_O_2_ concentration prior to spike/nM	% Recovery H_2_O_2_
V(IV)	1.0 nM	42	113
	2.0 nM	42	149
	20 nM	42	303
Fe(III)	3.5 nM	42	104
Fe(II)	130 pM	42	113
	330 pM	42	130
	330 pM	1.3	153
	2.6 nM	1.3	202

**Table 2:** Summary of deep ocean H_2_O_2_ data.

Transect	Depths*/m	H_2_O_2_ Range/nM	Mean H_2_O_2_ ( ± SD)/nM
Mediterranean, Sicily to Tunisia	100	0.61–3.1	1.5 ± 1.0
Mediterranean, Corsica to France	100	b/d–10	6.0 ± 4.2
	>1000	b/d–6.8	2.9 ± 2.2
Mediterranean, Balearics to Algeria	>1000	b/d–0.62	0.25 ± 0.27
Mediterranean, Sardinia to Sicily	100	b/d–9.9	1.7 ± 2.8
	>1000	b/d–6.1	0.55 ± 1.3
Mediterranean, Balearics to Spain	100	b/d–5.3	1.2 ± 2.1
S Atlantic, Angola Basin	100	0.46–9.6	3.6 ± 3.7
	>1000	b/d–4.6	0.90 ± 1.2
S Pacific, Peruvian upwelling**	100	2.8-9.0	5.0 ± 1.9
	>1000	b/d–2.1	1.1 ± 0.65

b/d ‘below detection limit’ (0.4–0.8 nM), SD standard deviation of the mean H_2_O_2_ including the possible H_2_O_2_ concentration range for samples below detection (i.e. any value lower than the detection limit for each section), *100 m depth inclusive of samples 90–110 m, >1000 m depths inclusive of samples from 950 m. Mean/range not shown for groupings with <5 datapoints. **S Pacific H_2_O_2_ data is likely to be an over-estimate due to the presence of interfering species (eg Fe(II)) and is included for indicative purposes only.
